# Interpretable deep learning approach for oral cancer classification using guided attention inference network

**DOI:** 10.1117/1.JBO.27.1.015001

**Published:** 2022-01-12

**Authors:** Kevin Chew Figueroa, Bofan Song, Sumsum Sunny, Shaobai Li, Keerthi Gurushanth, Pramila Mendonca, Nirza Mukhia, Sanjana Patrick, Shubha Gurudath, Subhashini Raghavan, Tsusennaro Imchen, Shirley T. Leivon, Trupti Kolur, Vivek Shetty, Vidya Bushan, Rohan Ramesh, Vijay Pillai, Petra Wilder-Smith, Alben Sigamani, Amritha Suresh, Moni Abraham Kuriakose, Praveen Birur, Rongguang Liang

**Affiliations:** a The University of Arizona, Wyant College of Optical Sciences, Tucson, Arizona, United States; b Mazumdar Shaw Medical Centre, Bangalore, Karnataka, India; c KLE Society Institute of Dental Sciences, Bangalore, Karnataka, India; d Mazumdar Shaw Medical Foundation, Bangalore, Karnataka, India; e Biocon Foundation, Bangalore, Karnataka, India; f Christian Institute of Health Sciences and Research, Dimapur, Nagaland, India; g University of California, Irvine, Beckman Laser Institute & Medical Clinic, Irvine, California, United States; h Cochin Cancer Research Center, Kochi, Kerala, India

**Keywords:** oral cancer, interpretable deep learning, guided attention inference network

## Abstract

**Significance:**

Convolutional neural networks (CNNs) show the potential for automated classification of different cancer lesions. However, their lack of interpretability and explainability makes CNNs less than understandable. Furthermore, CNNs may incorrectly concentrate on other areas surrounding the salient object, rather than the network’s attention focusing directly on the object to be recognized, as the network has no incentive to focus solely on the correct subjects to be detected. This inhibits the reliability of CNNs, especially for biomedical applications.

**Aim:**

Develop a deep learning training approach that could provide understandability to its predictions and directly guide the network to concentrate its attention and accurately delineate cancerous regions of the image.

**Approach:**

We utilized Selvaraju et al.’s gradient-weighted class activation mapping to inject interpretability and explainability into CNNs. We adopted a two-stage training process with data augmentation techniques and Li et al.’s guided attention inference network (GAIN) to train images captured using our customized mobile oral screening devices. The GAIN architecture consists of three streams of network training: classification stream, attention mining stream, and bounding box stream. By adopting the GAIN training architecture, we jointly optimized the classification and segmentation accuracy of our CNN by treating these attention maps as reliable priors to develop attention maps with more complete and accurate segmentation.

**Results:**

The network’s attention map will help us to actively understand what the network is focusing on and looking at during its decision-making process. The results also show that the proposed method could guide the trained neural network to highlight and focus its attention on the correct lesion areas in the images when making a decision, rather than focusing its attention on relevant yet incorrect regions.

**Conclusions:**

We demonstrate the effectiveness of our approach for more interpretable and reliable oral potentially malignant lesion and malignant lesion classification.

## 1 Introduction

Deep learning has become a powerful tool in solving image classification problems^1^ and has been widely used in medical image analysis.^2^ Convolutional neural networks (CNNs) show the potential for automated classification of different cancer lesions, such as lung cancer,^3^ skin cancer,^4^ and breast cancer.^5^ But the lack of interpretability and explainability makes CNNs less understandable.^6^ To push the envelope of CNN-based medical diagnostic methods, more rigorous neural network models and training methods must be developed to provide both visually interpretable and explainable analysis.^7^^–^^9^

Visual interpretability and attention maps for CNNs have been investigated for improving the robustness and accuracy.^10^^,^^11^ Class activation mapping (CAM)^12^ was developed to inject interpretability and explainability into decision-based deep learning models by highlighting the most discriminative region of an input image during classification. However, this approach requires modification of image classification CNN architectures, replacing fully connected layers with convolutional layers and global average pooling (GAP). Simonyan et al.^8^ introduced another approach to obtain visualization of image classification models by computing a class specific saliency map. By calculating the gradient of the class score with respect to the input image, these maps explain the regions of interest in the CNN’s classification decision. Selvaraju et al.^13^ developed a popular method for obtaining CNN attention maps called gradient-weighted CAM (Grad-CAM). This class-discriminative localization technique generates visual explanations for any CNN-based network without changing network architecture or re-training. As an effective way to inject visual explainability into the network’s classification decision, Grad-CAM can also be used to identify the limitations of the network. When attempting to classify a specific object or class, the CNN may incorrectly concentrate on other areas around the salient object, rather than the network’s attention focusing on the object to be recognized.^14^^–^^16^

While a CNN trained for oral potentially malignant lesion (OPML) and malignant lesion classification may correctly classify an image with lesions as suspicious/precancerous, it may in fact have its attention focused on a completely irrelevant region of the image upon making its decision. In these instances, the network has no incentive to focus solely on the correct subjects to be detected, leading to false positive classifications and poor generalization performance. To guide the network’s attention to the regions of interest, multiple methods have been proposed. Kim et al.^14^ presented a two-phase learning approach that combined the heat maps of the first and second networks to enhance object localization. Saleh et al.^17^ used the activations of the higher-level convolutional layers that were smoothed by a dense conditional random field to generate a more accurate foreground/background mask. Wei et al.^16^ proposed an approach that drove the classification network to sequentially discover new and complement object regions. Li et al.^18^ proposed the guided attention inference network (GAIN), a training architecture that took the attention maps generated by a weakly supervised deep learning network and provided direct guidance to the network in improving its own accuracy and completeness of its attention.

In this paper, we utilized Selvaraju et al.’s Grad-CAM to inject interpretability and explainability into a CNN. Furthermore, we adopted a two-stage training process with data augmentation techniques and GAIN. By utilizing the GAIN architecture, rather than simply training the CNN against classification loss, we jointly optimized the network’s classification and the network’s attention map in an end-to-end fashion. Further, by utilizing a combination of image labeled data and corresponding bounding box data we showed that our two-stage training method not only provided understandability to its predictions, but also directly guided the network to concentrate its attention and accurately delineate cancerous regions of the image. The network’s attention map will help us to actively understand what the network is focusing on and looking at during its decision-making process.

## 2 Methods

**Fig. 1:**
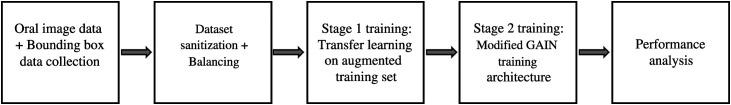
Block diagram of the proposed end-to-end deep learning approach for jointly optimizing OPML and malignant lesion classification and segmentation.

A block diagram of the entire experimental process is shown in Fig. 1. We used VGG19 as our base network for training. We first collected and obtained a rich dataset of oral images that were labeled and annotated with bounding boxes. These bounding boxes served to identify where the lesions were located. We removed out-of-focus images, rebalanced the data, and constructed a training set for stage 1 and stage 2 training. The third step was to conduct transfer learning on the deep CNN VGG19 coupled with further dataset augmentations on the prepared training set. The output was then passed through the GAIN training architecture with modified loss function. Finally, we analyzed the classification performance of the trained CNN, and compared its attention map against other state-of-the-art networks trained using only transfer learning.

## 2.1 Datasets

**Fig. 2:**
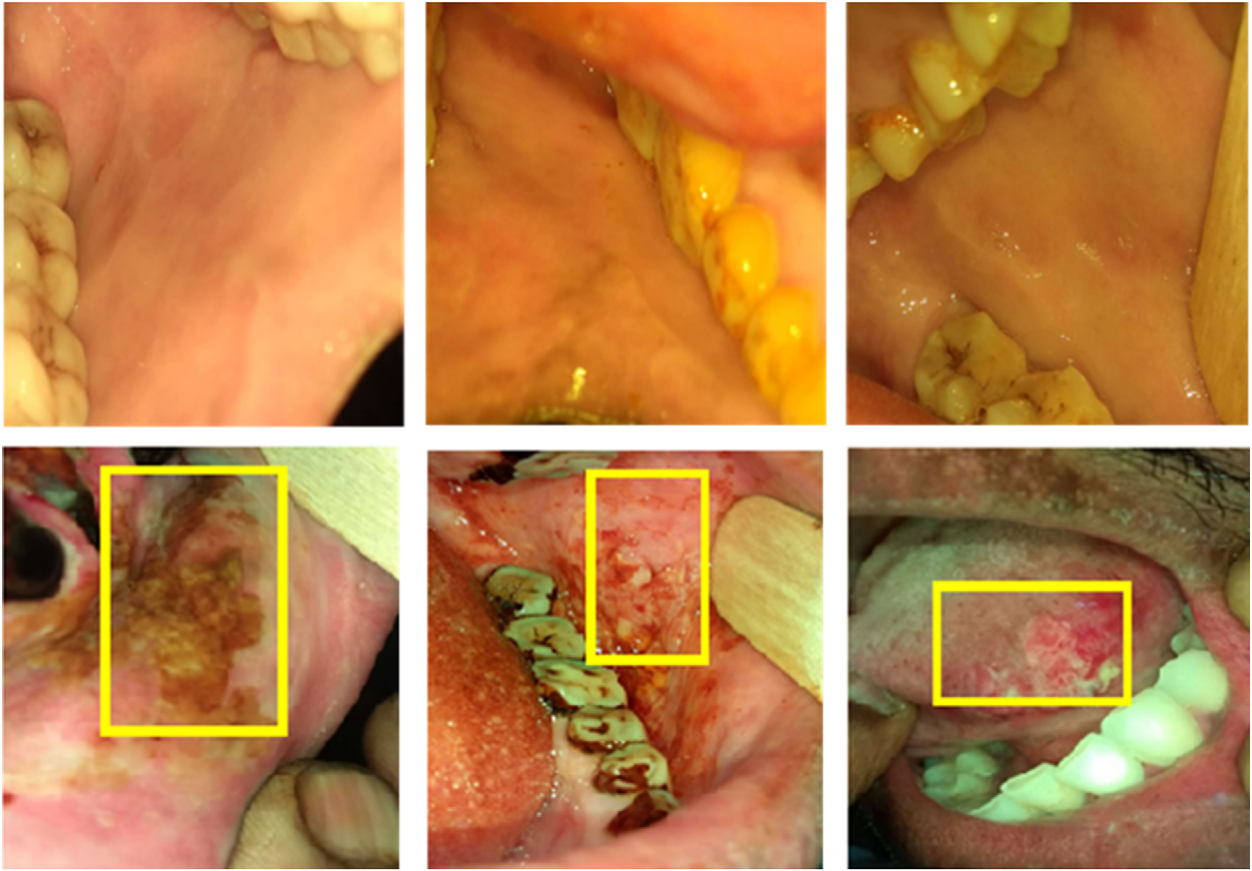
Examples of collected oral images, nonsuspicious cases (first row) and suspicious cases (second row). The red bounding box of the suspicious cases shows the lesion areas annotated by the specialists.

The dataset was obtained from patients attending the outpatient clinics of the Department of Oral Medicine and Radiology at KLE Society Institute of Dental Sciences, Head and Neck Oncology Department of Mazumdar Shaw Medical Center (MSMC), and Christian Institute of Health Sciences and Research (CIHSR), India. Institutional ethics committee approval was obtained from all participating hospitals, and written informed consents were collected from all subjects enrolled. Oral oncology specialists from MSMC, KLE, and CIHSR annotated all the images. Previous studies showed that oral oncology specialists’ interpretations had high accuracy with biopsy-confirmed cases.^19^ The dataset was captured using our customized oral cancer screening platform.^20^^,^^21^ Figure 2 shows some examples of nonsuspicious cases and suspicious cases with bounding boxes.

**Fig. 3:**
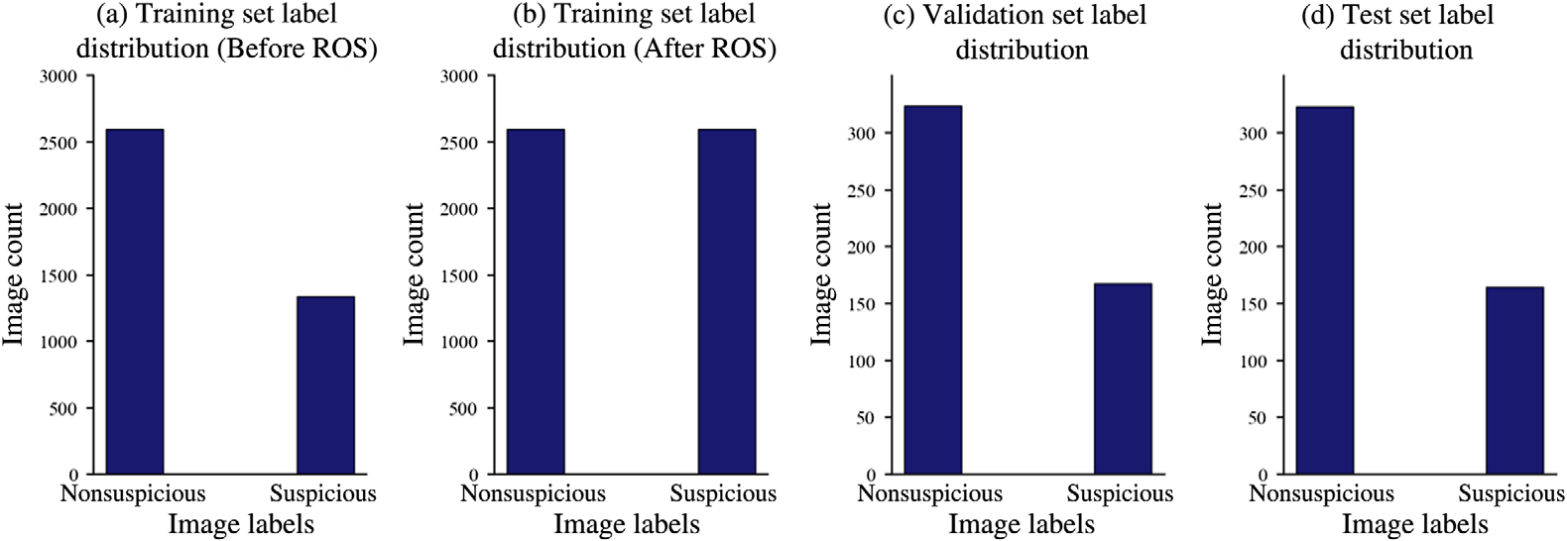
Data distributions of the training dataset (a) before and (b) after ROS, (c) validation dataset, and (d) testing dataset.

The dataset was cleaned in preparation for the training pipeline. The image data and its corresponding bounding box data were then subdivided into three dataset divisions of training, validation, and testing at 80%, 10%, and 10%, respectively. The image data were separated into two categories: “nonsuspicious,” which contains normal images, and “suspicious,” which contains OPML and malignant lesion images. As the training data were unbalanced between “suspicious” and “nonsuspicious,” with roughly 1200 less images in the “suspicious” class, we applied random oversampling (ROS) on the “suspicious” data in the training dataset. This process was achieved by randomly duplicating images in the training class labeled as “suspicious” with a replacement, until the two classes in the training dataset contained an equal number of images. Figure 3 shows the data distributions of the training dataset before and after ROS, validation dataset, and testing dataset.

### 2.2 Stage 1 Training: Transfer Learning on Augmented Training Set

Before training the network on the GAIN architecture, we used transfer learning on VGG19, replacing the last/fully connected layer with a new fully connected layer having two outputs corresponding to the two classes of “suspicious” and “nonsuspicious.” Typically, when training feed forward networks, such as CNN, its prediction error/loss is calculated and backpropagated through each layer of the network, thereby updating each layer’s corresponding weights to minimize the network’s overall error/loss. However, in transfer learning, the hidden layers of the original base VGG19 network provide an ideal initial condition for classification tasks. Therefore, to focus the learning/weight updates on the newly added last/fully connected layer rather than the existing hidden layers from VGG19, we multiplied the delta/change in weights and biases of this last layer by 10 during each training update, thereby focusing the CNN to learn to make changes to its newest layer instead of the existing layers that have been co-opted from VGG19.

Furthermore, to improve the generalizability performance of the CNN, during training we introduced slightly modified copies of the training images in a process referred to as data augmentation. Because OPML and malignant lesion are known to be flexible and stretchable as well as amorphous in shape, by introducing images with random scaling and shearing we were able to synthetically create these additional data points and improve both the classification and attention mining performance of the CNN. To further increase the dataset size, we also applied random rotations, reflections, translation, and padded any blank pixels at the edges of the image with RGB values of [128, 128, 128]. Thus, before an image was passed to the CNN during each training iteration, it was randomly rotated, scaled, reflected, sheared, translated, and padded by a random value chosen within the range of the corresponding augmentation ranges, as shown in Table 1.

**Table 1:** Dataset augmentation applied to stage 1 training.

Training data augmentations	
Random rotations	[−180 deg, 180 deg]
Random *X* scaling factor	[0.75*x*, 1.5*x*]
Random *Y* scaling factor	[0.75*x*, 1.5*x*]
Random *X* feflections	On
Random *Y* reflections	On
Random *X* shearing	[−20 pixels, 20 pixels]
Random *Y* shearing	[−20 pixels, 20 pixels]
Random *X* translation	[−5 pixels, 5 pixels]
Random *Y* translation	[−5 pixels, 5 pixels]
RGB fill value	[128, 128, 128]

**Fig. 4:**
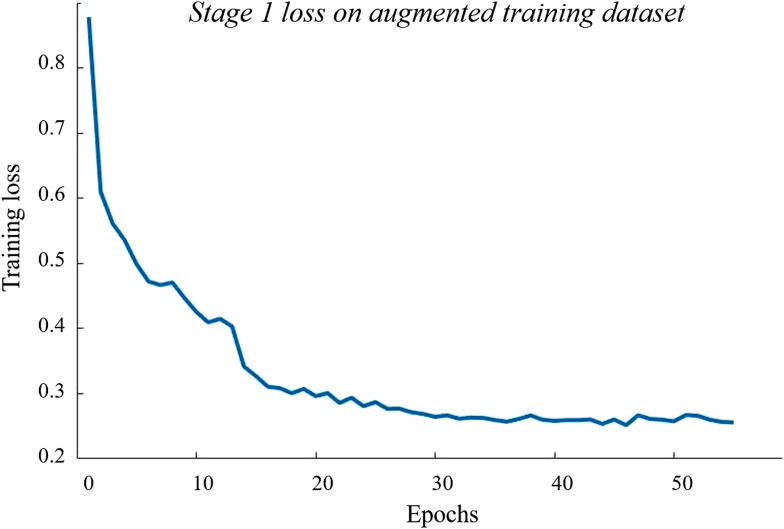
The loss curve during stage 1 training on the augmented training dataset.

In stage 1, the network was trained using stochastic gradient descent (SGD) for 55 epochs at a mini-batch size of 8 and a learning rate of 3e-4. We set the momentum to 0.9 and reduced the learning rate by a factor of 0.1 every 13 epochs. The training loss of stage 1 is shown in Fig. 4. Since our goal is to also improve the network’s attention accuracy with GAIN, we tuned the weight and bias learn rate factors from 10 to 2 in this stage to provide a more balanced learning rate across every CNN layer during the next training process.

### 2.3 Stage 2: GAIN Training Architecture

**Fig. 5:**
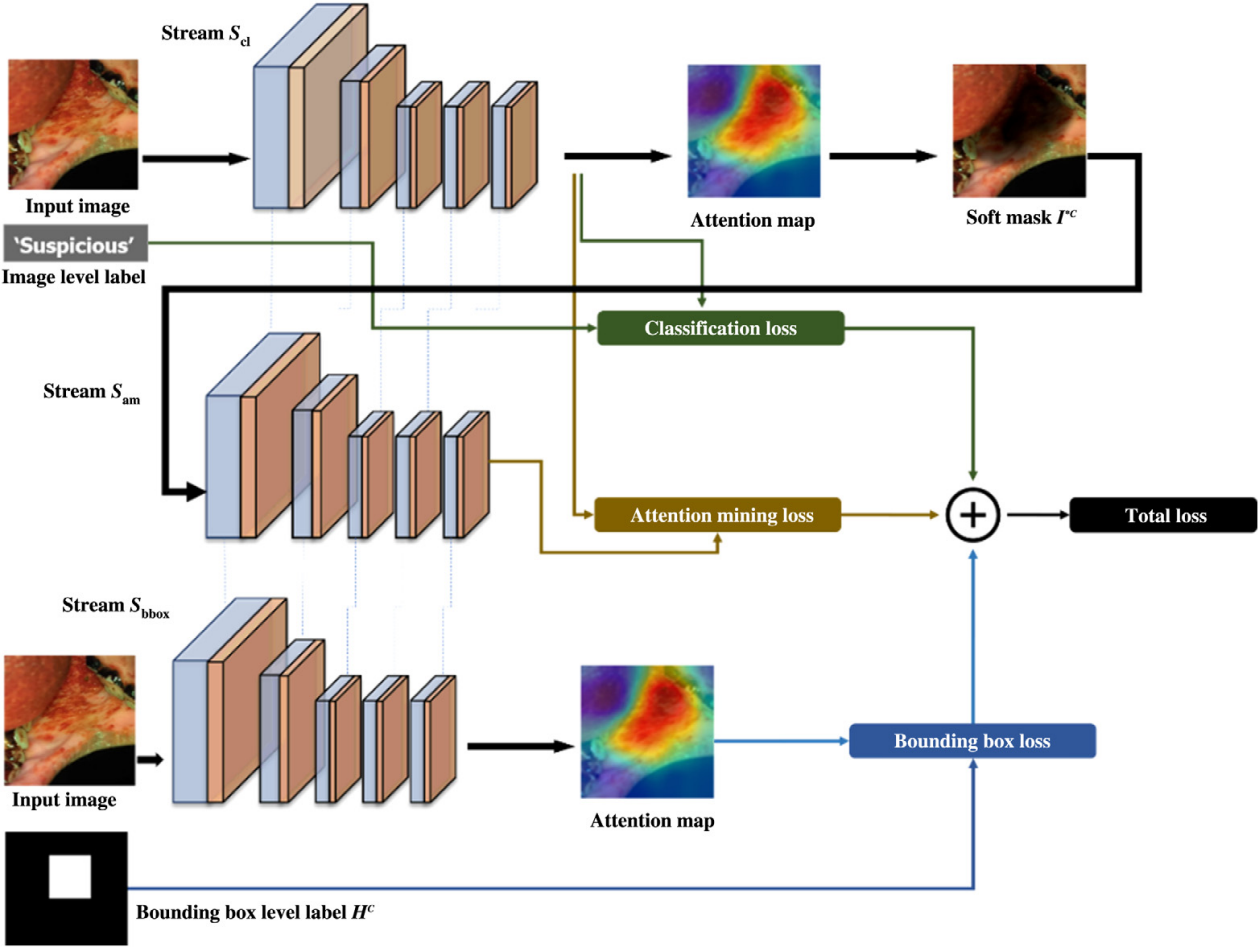
Utilizing image labels and bounding box data, a block diagram of the GAIN training architecture is shown for jointly optimizing the CNN’s classification and attention performance on OPML and malignant lesion data. The architecture consists of three streams of network training, classification stream $S_{\mathrm{cl}}$, attention mining stream $S_{\mathrm{am}}$, and bounding box stream $S_{\text{bbox}}$.

For stage 2, we trained the CNN utilizing the GAIN training architecture with modified loss function. Attention maps, such as those obtained by Grad-CAM, provide explainability and interpretability by indicating where in the input image the CNN is most focused on when making its classification decision. Conventionally, CNNs optimized using only classification loss during training produce attention maps that only highlight the most discriminative areas of interest in the input image. By adopting the GAIN training architecture, we jointly optimized the classification and segmentation accuracy by treating these attention maps as reliable priors to develop attention maps with more complete and accurate segmentation. In contrast to a typical single stream of CNN training optimized solely over classification, in using the GAIN architecture we utilized three streams of network training, sharing network parameters, and weights in concert. Weights were not updated during backpropagation until every stream was processed at the end of each training iteration. Figure 5 shows the algorithmic block diagram of the GAIN training process.

The first stream is the classification stream $S_{\mathrm{cl}}$. The $S_{\mathrm{cl}}$ stream’s goal performs canonical CNN training, optimizing solely over image labels for classification. For the $S_{\mathrm{cl}}$ stream, in our experiments we utilized SGD with a batch size of 1. The loss from this stream is referred to as classification loss $L_{\mathrm{cl}}$. From the classification output of each decision made during training, an attention map is generated indicating where the CNN is focused on. Similarly to Li et al.,^18^ we utilized Grad-CAM as the basis for generating attention maps during training. As a single input image I flowed through $S_{\mathrm{cl}}$, we calculated activation maps $f_{l,k}$ such that l indicated the l’th layer in the CNN and k indicated the corresponding unit in that layer. For each class c of the ground-truth classification labels, we calculated the gradient of the classification score $s_{c}$ with respect to activation maps $f_{l,k}$. GAP was then applied to the these gradients characterizing the neuron importance weights $w_{l,k}^{c}$, as shown in Eq. (1)

$$w_{l,k}^{c}=\mathrm{GAP}(\frac{\partial s^{c}}{\partial f_{l,k}}).$$ (1)

Finally, to obtain the corresponding attention map $A^{c}$ for the single input training image, we convolved the calculated weight matrix $w^{c}$ on the activation maps matrix $f_{l}$ and applied a ReLU operation on the output

$$A^{c}=\mathrm{ReLU}(\mathrm{conv}(f_{l},w^{c})).$$ (2)

The second stream is the attention mining stream $S_{\mathrm{am}}$. The goal of $S_{\mathrm{am}}$ is to train/enable the CNN to expand its outputted attention maps beyond the most discriminative regions learned in $S_{\mathrm{cl}}$ to more fully envelop and delineate the suspicious cancerous regions. To accomplish this task, a softmask was applied to the original input image I as a function of the $A^{c}$ output from $S_{cl}$. The resulting image, referred to as $I^{*C}$, portrayed the regions outside the network’s current main attention by weighting the masking more heavily on the areas of I corresponding to highest area of attention from $A^{c}$

$$I^{*c}=I-(T(A^{c})⊙I),$$ (3)

where ⊙ notation refers to element-wise multiplication and $T(A^{c})$ represents a thresholding function. The Sigmoid function is utilized in the $T(A^{c})$. For our experiments, σ is a hyperparameter matrix of size $A^{c}$ and w is an additional weighting hyperparameter meant to constrain and threshold $T(A^{c})$ between 0 and 1 such that it returns 1 when $A^{c}$ is larger than σ and 0 otherwise. However, in our experiments on the oral cancer dataset, we found setting the hyperparameter matrix σ to be a matrix of values containing 0.20, and setting weighting hyperparameter w to 3.0 yielded the most optimum results in training the CNN’s attention map on the cancerous lesions

$$T(A^{c})=\frac{1}{1+e^{-w(A^{c}-\sigma )}}.$$ (4)

Next, $I^{*C}$ was passed as input to stream $S_{\mathrm{am}}$, and the attention mining loss function $L_{\mathrm{am}}$ was calculated. As the objective here was to ensure the CNN’s attention map fully and concisely highlights the class of interest in input image I, $S_{\mathrm{am}}$ achieved this by training the CNN to ensure $I^{*C}$ fully masks out the same corresponding region utilizing a loss function that seeks to minimize the prediction score on $I^{*C}$ for each class c output: “suspicious” and “nonsuspicious.” However, in contrast to Li et al.’s GAIN $L_{\mathrm{am}}$ loss function, we found that reparametrizing our loss function as seen in Eq. (5) produced results that led to more optimum convergence

$$L_{\mathrm{am}}=|0.5-\underset{c}{\sum }s^{c}(I^{*C})|.$$ (5)

The third stream $S_{\text{bbox}}$ utilizes the additional bounding box dataset. This dataset was hand curated by the medical team to indicate the boxed regions of each image where suspicious lesions were located. The goal of this stream was to provide additional supervision/training on the CNN attention maps to further enforce the accuracy of where they should focus. Instead of using Li et al.’s GAIN bounding box extension ${\mathrm{GAIN}}_{\mathrm{ext}}^{b}$, we utilized Li et al.’s GAIN pixel-level supervision extension ${\mathrm{GAIN}}_{\mathrm{ext}}^{p}$ and found it to show superior performance on the bounding box dataset. The following loss function $L_{\text{bbox}}$ was utilized

$$L_{\text{bbox}}=\;\frac{1}{n}\underset{c}{\sum }{\left(A^{c}-H^{C}\right)}^{2},\;$$ (6)

where n is the number of class labels, $A^{c}$ is the attention map for image I output from $S_{\mathrm{cl}}$, and $H^{C}$ is the bounding box ground-truth produced by creating a binarized matrix the size I such that values inside the bounding box were set to 1 and values outside the box were set to 0. For images whose ground-truth label was “nonsuspicious” and thus did not contain any lesions, we let $H^{C}$ be a matrix of size $A^{c}$ with values all equal to 0.

Finally, to jointly optimize across all three streams, a combined loss function $L_{\text{total}}$ was constructed

$$L_{\text{total}}=\zeta L_{\mathrm{cl}}+\alpha L_{\mathrm{am}}+\beta L_{\text{bbox}}.$$ (7)

Weighting parameters ζ, α, and β were chosen as hyperparameters to tune the weighting of each stream in achieving the joint optimization/minimization of the total loss $L_{\text{total}}$. Optimum performance in our experimentation was found by setting ζ=0.25, α=0.5, and β=360 such that the maximum value of $\zeta L_{\mathrm{cl}}$ over every iteration never surpassed 2.0, the maximum value of α$L_{\mathrm{am}}$ over every iteration never surpassed 0.5, and the average value of β$L_{\text{bbox}}$ over every iteration average was roughly 0.5. We did not use the original hyperparameters from the original GAIN paper, as numerical optimization is highly data dependent, our network hyperparameters ζ=0.25, α=0.5, and β=360 were chosen intentionally as to slowly yet swiftly approach the ideal solution.

We utilized SGD during stage 2 training with a learning rate of $10^{-4}$ and a mini-batch size of 1. With the set hyperparameters corresponding to classification loss, attention mining loss, and bounding box loss, we ran the GAIN training architecture for 5 epochs. Training was run on University of Arizona’s High Powered Computing (HPC) platform and terminated after 26 h.

## 3 Results and Discussion

**Fig. 6:**
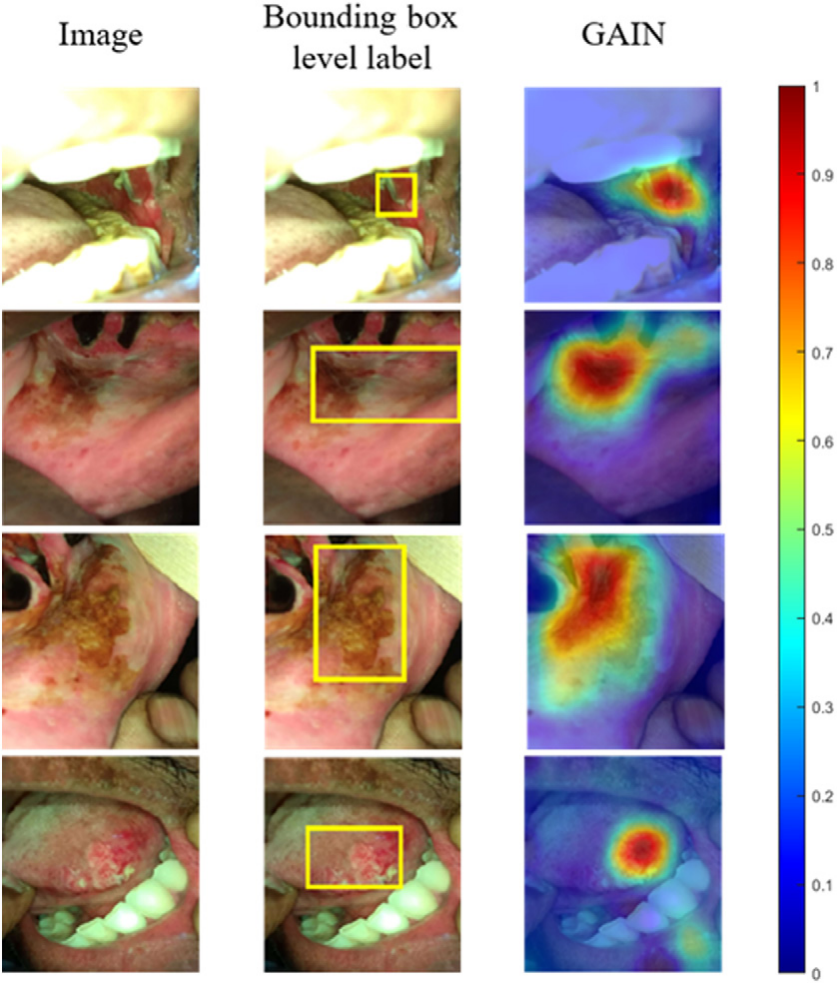
Test dataset images correctly classified as “suspicious.” The first column shows the images input into the CNN. Bounding box annotated images show in the second column, indicating the location of the OPML and malignant lesion as annotated by the medical team. Attention map (third column) output by the CNN explaining/segmenting the region of the image the network’s attention is focused on when making its classification decision; red indicates area of highest attention and blue indicates area of lowest attention.

Classification accuracy of the proposed network after stage 2 training was 86.38% (78.9% sensitivity/90.5% specificity) on the validation dataset and 84.84% (76.6% sensitivity/89.3% specificity) on the test dataset, respectively. The classification accuracy of the network after stage 1 training reached 84.55% accuracy (74.7% sensitivity/90.5% specificity) on the validation dataset and 83.81% accuracy (74.4% sensitivity/89.1% specificity) on the test dataset, respectively. Figure 6 shows the typical results of the GAIN trained network, the network’s attention maps aptly highlighted the correct lesion areas in the image when predicting the result. The GAIN trained network’s attention maps show the ability to acquire more detailed borders of the lesion regions than the original bounding box labels used for training. The results demonstrate that the GAIN trained network can accurately and effectively envelop and delineate the suspicious lesions as expected.

**Fig. 7:**
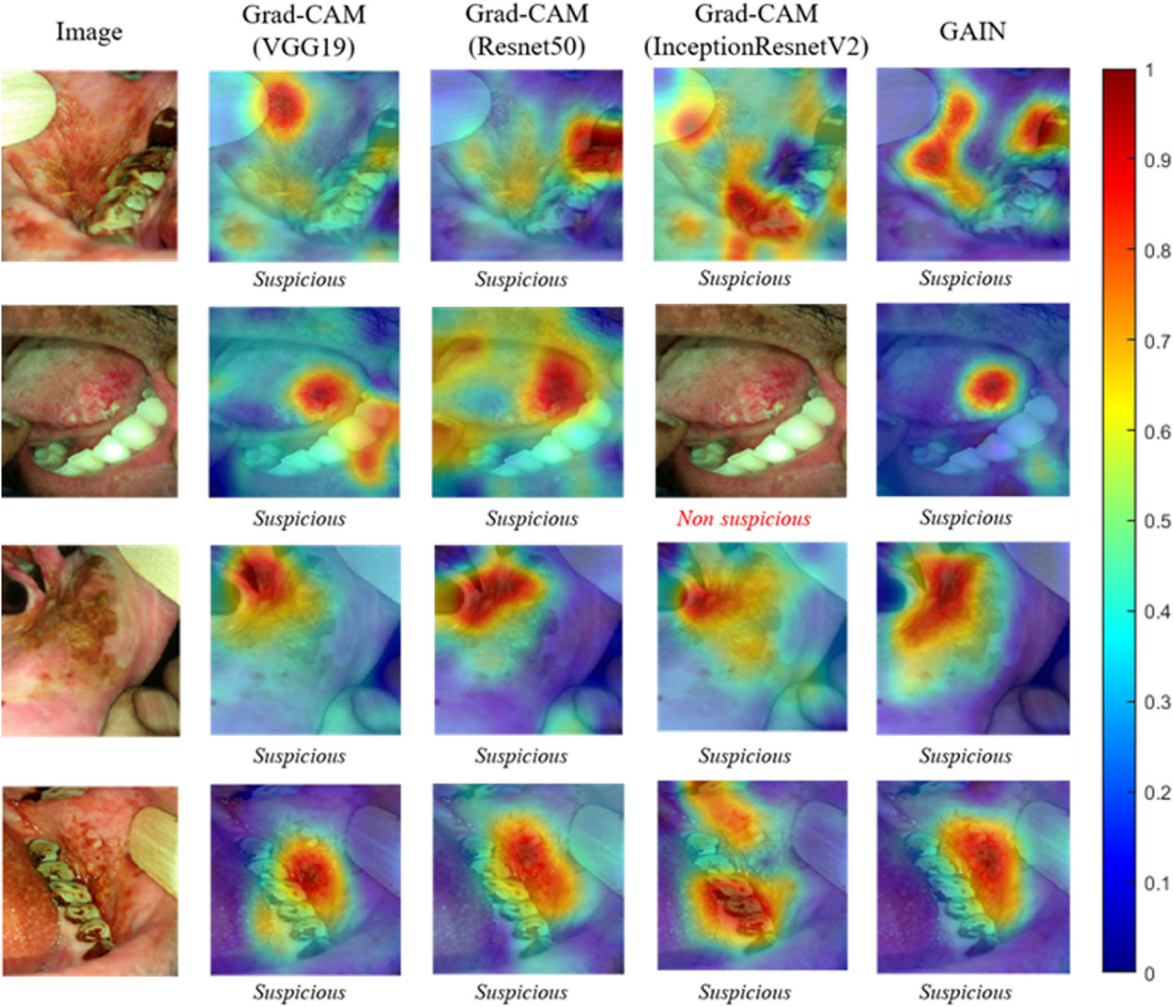
Comparison between the output attention map generated by the proposed method and other conventional transfer learning trained networks: VGG19, Resnet50, and Inceptionresnetv2. Areas highlighted in red indicate the highest attention area when the CNN made its decision, and blue indicates the areas of lowest attention.

Additionally, we conducted comparative studies of the proposed method with networks trained with conventional transfer learning and visualized with Grad-CAM. We used VGG19, Resnet50, and InceptionResnetV2 pretrained from ImageNet as the basic networks for the comparison; these networks were canonically trained only on image labels. For comparison purposes, we used the same hyperparameter settings from stage 1 with an additional 5 epochs of training to match the total learning time of our GAIN trained network. Using SGD for 60 epochs at a mini-batch size of 8 and at a learning rate of 3e-4, we set the momentum to 0.9 and reduced the learning rate by a factor of 0.1 every 13 epochs. The comparison results are shown in Fig. 7.

The examples in Fig. 7 show that the CNN’s attention mapping/segmentation performance in fully encompassing and delineating the cancerous lesions excelled over other networks trained with conventional transfer learning and visualized with Grad-CAM. For example, for the first row of Fig. 7, while each of the networks correctly classified the image as “suspicious,” Resnet50’s attention was incorrectly focused on stained teeth, and both VGG19 and Inceptionresnetv2 conflated the presence of the tongue depressor as indicating the suspicious presence of cancer. In the second row, InceptionresnetV2 misclassified the image, and VGG19 and Resnet50’s attention maps, in addition to the cancerous lesion, also activated other incorrect areas. In the third row, compared with the attention map generated with the proposed method, the attention maps of networks trained with conventional transfer learning did not completely delineate the lesion areas. A similar situation also appears in the fourth row.

The attention maps that focused on the tongue depressor and stained teeth were likely due to some of our dataset’s “suspicious” images having had the tongue depressor and stained teeth in the image. The correlation between “suspicious” lesions and tongue depressor might be because some patients with cancerous or potentially cancerous lesions cannot open their mouth fully, and the medical team used the tongue depressor to help take a clear photo. In addition, many OPML and malignant lesion patients in India were as a result of tobacco chewing, which could also cause staining of their teeth. This might explain the correlation between “suspicious” lesions and stained teeth. With the conventional training process, there is no incentive in training to focus the network’s attention on only the lesion area.

## 4 Conclusion

In this paper, we proposed a two stage training process involving transfer learning and data augmentation in stage 1 and adoption of GAIN training architecture in stage 2, to improve the accuracy of classification performance, inject visual explainability, and guide the trained CNN’s attention to the lesion areas in input images. Utilizing image labels and bounding box data, we developed the CNN by jointly optimizing the accuracy of the classification, attention maps, and attention mining in an end-to-end manner.

We succeeded in validating this process by reporting the CNN’s classification performance on the test set and evaluating the output attention maps. We compared our method results with networks trained with conventional transfer learning and visualized with Grad-CAM. These results show that the proposed method could guide the trained neural network to highlight and focus its attention on the correct lesion areas in the images when making a decision, rather than focusing its attention on relevant yet incorrect regions (such as a tongue depressor). The proposed method effectively improves the explainability and interpretability of the OPML and malignant lesion classification deep learning model and potentially improves the generalization performance by encouraging the network to not only correctly classify images but also develop a deeper understanding of what specifically in the image contributes to correct classification. The method increases the potential of deep learning models to be integrated into actual real-world medical procedures.

In the future, we will generate a more accurate pixel-level labeled (instead of bounding box) dataset to further optimize the performance of networks trained with the GAIN architecture. We believe it would more accurately guide the network to focus its attention on the correct lesion areas.
